# The Rate of Catheter-Related Infections using Metal Coated Central Venous Catheters; a Letter to Editor

**Published:** 2019-06-25

**Authors:** Seyed Hossein Ardehali, Mona Jahangirian, Alireza Fatemi

**Affiliations:** 1 Department of Anesthesiology and Critical Care, Shohadaye Tajrish Hospital, Shahid Beheshti University of Medical Sciences, Tehran, Iran.; 2 Internal Medicine Department, Shohadaye Tajrish Hospital, Faculty of Medicine, Shahid Beheshti University of Medical Sciences, Tehran, Iran.; 3 Men's Health and Reproductive Health Research Center, Shahid Beheshti University of Medical Sciences, Tehran, Iran.

**Keywords:** Central venous catheters, cross infection, infection control, intensive care units, sepsis

**Dear editor**

Blood infections due to intravenous catheters make up about 10% – 15% of hospital infections (1). In 2009, Centers for Disease Control and Prevention (CDC) reported the rate of blood infections related with using central venous catheter in the intensive care unit (ICU) to be 1.65 in 1000 catheters per day (2). Mortality due to infections related to central venous catheters has been reported to be between 12% and 25% in different studies. These infections have increased the duration of hospitalization by 12 days (3, 4).

Different approaches have been proposed for reducing these infections, among these approaches using aseptic methods, preventive antibiotics, disposable tools, and training the staff can be pointed out (5-7). Among the methods considered in this regard is using catheters coated with antiseptic agents, antibiotics, and metals such as silver and platinum (8). Some studies have suggested using these methods for reducing the mentioned infections and their consequences; however, their use is not currently agreed upon and their effect on reducing the infections caused by intravenous tools is still being studied.

Recently, these tools have become available in Iran and since there is limited or insufficient experience working with them, the authors of the present letter designed a comparative study aiming to evaluate the role of central venous catheters coated with metals such as gold, silver, and palladium in the rate of catheter-related infections.

In this study, 138 patients with the mean age of 60.62 ± 20.13 (17 – 97) years were randomly divided into 2 groups receiving either coated or non-coated central venous catheters, and then studied (58% male). The 2 groups were similar regarding sex (p = 0.730) and age (p = 0,409) distribution. 35 patients in the coated group and 42 in the non-coated group developed fever. Finally, 22 (15.9%) cases of infection due to catheter were observed, 8 (36.4%) of which were in the coated group and 14 (63.6%) were in the non-coated group (p = 0.163).

In culture, out of the 8 cases in the coated group, 4 cases of Acinetobacter baumannii, 2 cases of klebsiella pneumoniae, 1 case of escherichia coli, and 1 case of staphylococcus aureus grew. The Gram negative organisms found were all resistant to fluoroquinolone family, penicillin, aminoglycosides, third and fourth generation cephalosporins, carbapenems, and colistin. Meanwhile, in culture of the 14 infectious cases found in the non-coated group, 10 cases had acinetobacter baumannii, 1 case had klebsiella pneumoniae, and in 3 cases escherichia coli had grown. All the mentioned organisms were resistant to fluoroquinolone family, penicillin, aminoglycosides, third and fourth generation cephalosporins, carbapenems, and colistin. In the end, 4 (50%) cases out of the 8 infected cases in the coated catheter group and 8 (57.81%) cases out of the 14 in the non-coated group died (p > 0.05).

Based on the results obtained in this study, it seems that despite the decrease in the number of infected cases when metal coated catheters were used, this difference is not statistically significant. The same finding is true when comparing the mortality rates of the 2 groups.

Therefore, considering the higher cost imposed on the patients for using these tools, more thought should be given to using them. Of course, there is a need for more studies with more accuracy and control groups before the results of the present study can be generalized.
